# 2-Chloro-4-iodo­aniline

**DOI:** 10.1107/S1600536808036076

**Published:** 2008-11-08

**Authors:** Yun-Hua Xu, Can Wang, Fanqi Qu

**Affiliations:** a School of Science, Beijing Jiaotong University, Beijing 100044, People’s Republic of China; b College of Chemistry and Molecular Sciences, Wuhan University, Wuhan, Hubei 430072, People’s Republic of China

## Abstract

The title dihaloaniline, C_6_H_5_ClIN, shows no significant hydrogen bonds nor the commonly observed I⋯I inter­actions in the crystal structure, although an amino group and an I atom are available for such contacts. The crystal structure is stabilized by weak inter­actions involving the amine functionality as donor group and N or halogen atoms as acceptors.

## Related literature

The title compound was first synthesized 90 years ago (Dains *et al.*, 1918 ▶). For structures of halogenated anilines, see: Cox (2001 ▶); Dey *et al.* (2003 ▶); Dou *et al.* (1993 ▶); Fukuyo *et al.* (1982 ▶); Goubitz *et al.* (2001 ▶); Parkin *et al.* (2005 ▶); Sakurai *et al.* (1963 ▶).
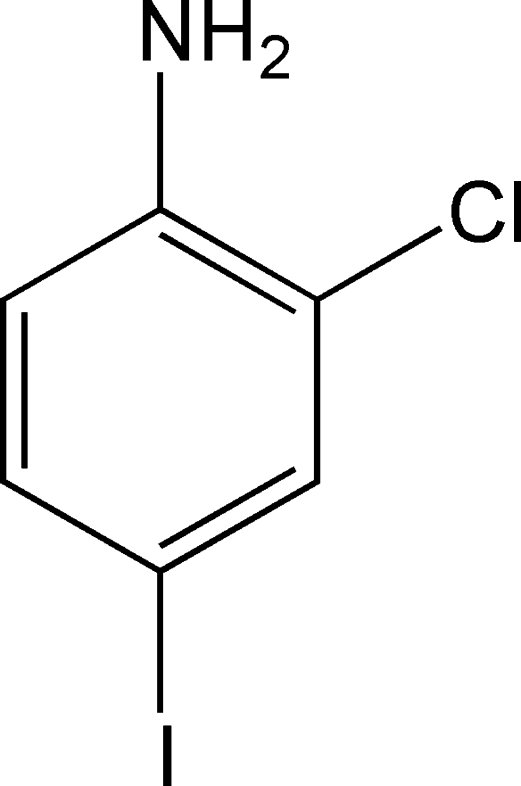


## Experimental

### 

#### Crystal data

C_6_H_5_ClIN
*M* *_r_* = 253.46
Orthorhombic, 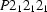

*a* = 5.6277 (2) Å
*b* = 8.7859 (3) Å
*c* = 14.9217 (5) Å
*V* = 737.79 (4) Å^3^
*Z* = 4
Mo *K*α radiation
μ = 4.61 mm^−1^
*T* = 90.0 (2) K
0.22 × 0.15 × 0.10 mm

#### Data collection

Nonius KappaCCD diffractometer
Absorption correction: multi-scan (*SCALEPACK*; Otwinowski & Minor, 1997 ▶) *T*_min_ = 0.424, *T*_max_ = 0.630
5635 measured reflections
1696 independent reflections
1587 reflections with *I* > 2σ(*I*)
*R*_int_ = 0.033

#### Refinement

*R*[*F* ^2^ > 2σ(*F* ^2^)] = 0.024
*wR*(*F* ^2^) = 0.046
*S* = 1.14
1696 reflections
89 parameters
1 restraint
H atoms treated by a mixture of independent and constrained refinement
Δρ_max_ = 1.18 e Å^−3^
Δρ_min_ = −0.76 e Å^−3^
Absolute structure: Flack (1983 ▶), 681 Friedel pairs
Flack parameter: −0.03 (3)

### 

Data collection: *COLLECT* (Nonius, 2002 ▶); cell refinement: *SCALEPACK* (Otwinowski & Minor, 1997 ▶); data reduction: *DENZO–SMN* (Otwinowski & Minor, 1997 ▶); program(s) used to solve structure: *SHELXS97* (Sheldrick, 2008 ▶); program(s) used to refine structure: *SHELXL97* (Sheldrick, 2008 ▶); molecular graphics: *SHELXTL* (Sheldrick, 2008 ▶); software used to prepare material for publication: *SHELXL97* and local procedures.

## Supplementary Material

Crystal structure: contains datablocks global, I. DOI: 10.1107/S1600536808036076/bh2201sup1.cif

Structure factors: contains datablocks I. DOI: 10.1107/S1600536808036076/bh2201Isup2.hkl

Additional supplementary materials:  crystallographic information; 3D view; checkCIF report

## Figures and Tables

**Table 1:** Hydrogen-bond geometry (Å, °)

*D*—H⋯*A*	*D*—H	H⋯*A*	*D*⋯*A*	*D*—H⋯*A*
N1—H1N⋯N1^i^	0.82 (3)	2.61 (3)	3.359 (4)	153 (4)
N1—H1N⋯Cl1^ii^	0.82 (3)	2.94 (4)	3.515 (4)	129 (4)
N1—H2N⋯I1^iii^	0.81 (3)	3.16 (3)	3.807 (4)	139 (4)

Symmetry codes: (i) 
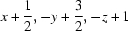
; (ii) 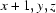; (iii) 
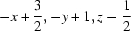
.

**Fig. 1.**
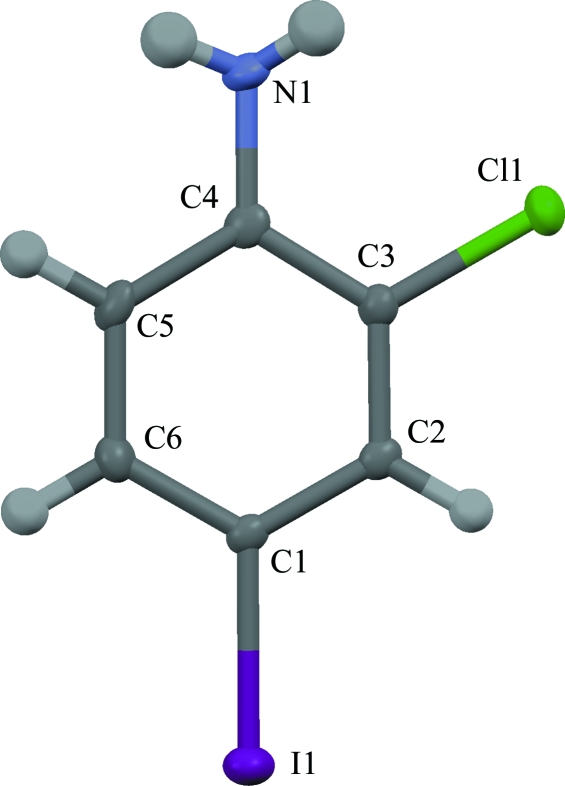
The molecular structure of (I), with displacement ellipsoids drawn at the 50% probability level (arbitrary spheres for the H atoms).

**Fig. 2.**
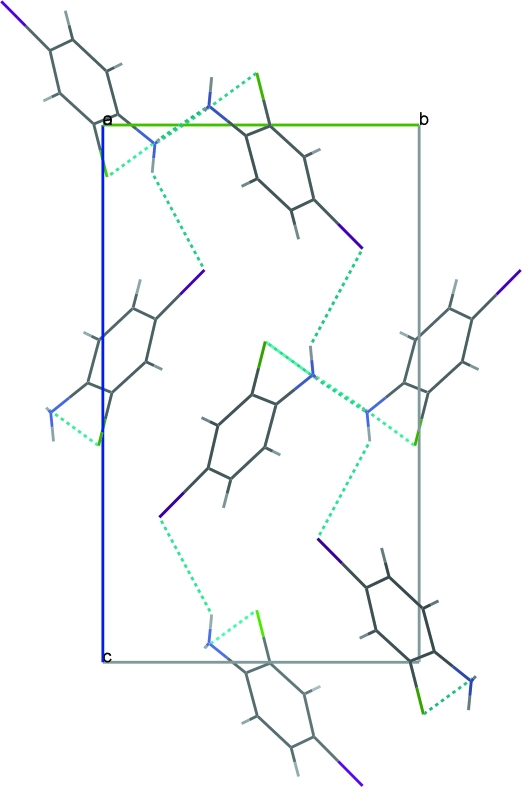
A packing diagram of (I) down the a axis.

## supplementary crystallographic information

### Comment

Although structurally simple and readily available, few crystal structures of dihaloanilines have been measured. A total of 10 structures were found in the 2007 CSD; the refcodes are CAJWEQ, CAJWEQ01 (Goubitz *et al.*, 2001), DCHLAN, DCHLAN01 (Sakurai *et al.*, 1963), KUMTER (Cox, 2001), WEMDAT, WEMDEX, WEMDIB, WEMDOH, WEMDUN (Dou *et al.*, 1993). 2-Chloro-4-iodoaniline, (I), an aniline with two different halogen substituents, was first synthesized 90 years ago (Dains *et al.*, 1918), yet its crystal structure is reported here for the first time.

The asymmetric unit contains one molecule (Fig. 1). The N atom is not coplanar with the aromatic ring; H atoms of the amino group are also out of the halogenated benzene ring, but in the opposite direction to that of the N atom. So, the C(Ar)NH_2_ group has a pyramidal shape. This is similar to the structure of aniline at 252 K (Fukuyo *et al.*, 1982), 2-iodoaniline at 100 K (Parkin *et al.*, 2005) and 4-iodoaniline at 203 K (Dey *et al.*, 2003).

Despite the presence of amino, chloro and iodo groups, no classic interactions associated with them, such as hydrogen bonds, Cl···Cl, or I···I contacts were observed in the crystal structure of (I). Instead, weak interactions such as N—H···N, N—H···I, and N—H···Cl are found to provide stability to the crystal (Fig. 2).

### Experimental

The compound was purchased from TCI America Laboratory Chemicals as colorless block crystals suitable for single-crystal X-ray diffraction measurement.

### Refinement

H atoms were found in a difference map and those on the aromatic ring subsequently placed in idealized positions with C—H distances of 0.95 Å and isotropic displacement parameters equal to 1.2*U*_eq_ of the carrier C atom. Amine H atoms H1N and H2N were refined freely but were restrained to converge to the same N—H bond lengths, with a standard deviation of 0.02 Å. Isotropic displacement parameters for H1N and H2N were computed as 1.5*U*_eq_(N1)

### Crystal data

**Table:**

C_6_H_5_ClIN	*F*_000_ = 472
*M**_r_* = 253.46	*D*_x_ = 2.282 Mg m^−^^3^
Orthorhombic, *P*2_1_2_1_2_1_	Mo *K*α radiation λ = 0.71073 Å
Hall symbol: P 2ac 2ab	Cell parameters from 1019 reflections
*a* = 5.6277 (2) Å	θ = 1.0–27.5º
*b* = 8.7859 (3) Å	µ = 4.61 mm^−^^1^
*c* = 14.9217 (5) Å	*T* = 90.0 (2) K
*V* = 737.79 (4) Å^3^	Rounded block, colourless
*Z* = 4	0.22 × 0.15 × 0.10 mm

### Data collection

**Table:**

Nonius KappaCCD diffractometer	1696 independent reflections
Radiation source: fine-focus sealed tube	1587 reflections with *I* > 2σ(*I*)
Monochromator: graphite	*R*_int_ = 0.033
Detector resolution: 18 pixels mm^-1^	θ_max_ = 27.5º
*T* = 90.0(2) K	θ_min_ = 2.7º
ω scans at fixed χ = 55°	*h* = −7→7
Absorption correction: multi-scan(SCALEPACK; Otwinowski & Minor, 1997)	*k* = −11→11
*T*_min_ = 0.424, *T*_max_ = 0.630	*l* = −19→19
5635 measured reflections	

### Refinement

**Table:**

Refinement on *F*^2^	Hydrogen site location: inferred from neighbouring sites
Least-squares matrix: full	H atoms treated by a mixture of independent and constrained refinement
*R*[*F*^2^ > 2σ(*F*^2^)] = 0.024	*w* = 1/[σ^2^(*F*_o_^2^) + (0.*P*)^2^ + 0.4678*P*] where *P* = (*F*_o_^2^ + 2*F*_c_^2^)/3
*wR*(*F*^2^) = 0.046	(Δ/σ)_max_ = 0.001
*S* = 1.14	Δρ_max_ = 1.18 e Å^−^^3^
1696 reflections	Δρ_min_ = −0.76 e Å^−^^3^
89 parameters	Extinction correction: SHELXL97, Fc^*^=kFc[1+0.001xFc^2^λ^3^/sin(2θ)]^-1/4^
1 restraint	Extinction coefficient: 0.0021 (3)
Primary atom site location: structure-invariant direct methods	Absolute structure: Flack (1983), 681 Friedel pairs
Secondary atom site location: difference Fourier map	Flack parameter: −0.03 (3)

### Fractional atomic coordinates and isotropic or equivalent isotropic displacement parameters (Å^2^)

**Table:**

	*x*	*y*	*z*	*U*_iso_*/*U*_eq_	
I1	0.48590 (4)	0.17882 (2)	0.730162 (15)	0.01928 (9)	
Cl1	0.46148 (17)	0.51349 (10)	0.40305 (6)	0.0194 (2)	
N1	0.9067 (6)	0.6647 (5)	0.4646 (2)	0.0188 (8)	
H1N	1.048 (5)	0.679 (5)	0.474 (3)	0.028*	
H2N	0.882 (7)	0.656 (5)	0.411 (2)	0.028*	
C1	0.6370 (6)	0.3328 (4)	0.6386 (2)	0.0133 (7)	
C2	0.5194 (7)	0.3639 (3)	0.5594 (2)	0.0138 (7)	
H2	0.3764	0.3121	0.5448	0.017*	
C3	0.6128 (6)	0.4711 (4)	0.5018 (2)	0.0145 (8)	
C4	0.8253 (6)	0.5480 (4)	0.5199 (3)	0.0152 (8)	
C5	0.9422 (6)	0.5117 (4)	0.5999 (2)	0.0166 (8)	
H5	1.0875	0.5611	0.6142	0.020*	
C6	0.8494 (7)	0.4049 (4)	0.6588 (3)	0.0163 (8)	
H6	0.9312	0.3812	0.7127	0.020*	

### Atomic displacement parameters (Å^2^)

**Table:**

	*U*^11^	*U*^22^	*U*^33^	*U*^12^	*U*^13^	*U*^23^
I1	0.02354 (13)	0.01764 (13)	0.01666 (14)	−0.00271 (12)	0.00379 (13)	0.00106 (9)
Cl1	0.0189 (5)	0.0231 (4)	0.0163 (4)	0.0003 (4)	−0.0036 (4)	−0.0007 (3)
N1	0.0163 (16)	0.0192 (18)	0.0208 (18)	−0.0046 (15)	0.0035 (15)	0.0025 (15)
C1	0.0136 (16)	0.0101 (17)	0.0161 (19)	0.0001 (15)	0.0027 (15)	−0.0029 (16)
C2	0.0142 (18)	0.0107 (15)	0.0165 (17)	0.0005 (16)	0.003 (2)	−0.0046 (13)
C3	0.0134 (18)	0.0133 (17)	0.0169 (19)	0.0020 (16)	−0.0006 (16)	−0.0017 (16)
C4	0.0121 (18)	0.0129 (18)	0.020 (2)	0.0043 (15)	0.0034 (16)	−0.0031 (17)
C5	0.0096 (18)	0.0154 (17)	0.025 (2)	0.0007 (14)	0.0002 (16)	−0.0053 (15)
C6	0.0172 (19)	0.0182 (19)	0.0133 (18)	0.0019 (16)	−0.0003 (16)	−0.0034 (16)

### Geometric parameters (Å, °)

**Table:**

I1—C1	2.103 (4)	C2—C3	1.379 (5)
Cl1—C3	1.742 (4)	C2—H2	0.9500
N1—C4	1.394 (5)	C3—C4	1.400 (5)
N1—H1N	0.82 (3)	C4—C5	1.400 (5)
N1—H2N	0.81 (3)	C5—C6	1.387 (5)
C1—C2	1.382 (5)	C5—H5	0.9500
C1—C6	1.386 (5)	C6—H6	0.9500
			
C4—N1—H1N	110 (3)	C4—C3—Cl1	118.6 (3)
C4—N1—H2N	117 (3)	N1—C4—C5	121.2 (3)
H1N—N1—H2N	110 (5)	N1—C4—C3	121.4 (3)
C2—C1—C6	120.6 (3)	C5—C4—C3	117.2 (3)
C2—C1—I1	119.3 (3)	C6—C5—C4	121.1 (3)
C6—C1—I1	120.1 (3)	C6—C5—H5	119.4
C3—C2—C1	119.0 (3)	C4—C5—H5	119.4
C3—C2—H2	120.5	C1—C6—C5	119.7 (3)
C1—C2—H2	120.5	C1—C6—H6	120.1
C2—C3—C4	122.3 (3)	C5—C6—H6	120.1
C2—C3—Cl1	119.1 (3)		
			
C6—C1—C2—C3	−1.9 (5)	Cl1—C3—C4—C5	179.8 (3)
I1—C1—C2—C3	176.5 (2)	N1—C4—C5—C6	174.2 (3)
C1—C2—C3—C4	1.2 (5)	C3—C4—C5—C6	−0.5 (5)
C1—C2—C3—Cl1	−178.6 (3)	C2—C1—C6—C5	1.5 (5)
C2—C3—C4—N1	−174.7 (3)	I1—C1—C6—C5	−176.9 (2)
Cl1—C3—C4—N1	5.1 (5)	C4—C5—C6—C1	−0.3 (5)
C2—C3—C4—C5	0.0 (5)		

### Hydrogen-bond geometry (Å, °)

**Table:**

*D*—H···*A*	*D*—H	H···*A*	*D*···*A*	*D*—H···*A*
N1—H1N···N1^i^	0.82 (3)	2.61 (3)	3.359 (4)	153 (4)
N1—H1N···Cl1^ii^	0.82 (3)	2.94 (4)	3.515 (4)	129 (4)
N1—H2N···I1^iii^	0.81 (3)	3.16 (3)	3.807 (4)	139 (4)

Symmetry codes: (i) *x*+1/2, −*y*+3/2, −*z*+1; (ii) *x*+1, *y*, *z*; (iii) −*x*+3/2, −*y*+1, *z*−1/2.
